# Analysis of an acute care surgery service performing temporal artery biopsy for suspected giant cell arteritis

**DOI:** 10.1016/j.sopen.2026.06.006

**Published:** 2026-06-29

**Authors:** Tynan H. Friend, Madeline Goosman, Abigail Hancock, Adam Aluisio, Nelson Oliveira, Manuel Silva, Emanuel. Dias, Daithi S. Heffernan, Andrew H. Stephen

**Affiliations:** a Division of Trauma and Surgical Critical Care, Department of Surgery, Warren Alpert Medical School of Brown University, Rhode Island Hospital, Providence, RI 02903; b Department of Emergency Medicine, Warren Alpert Medical School of Brown University, Rhode Island Hospital, Providence, RI 02903; c Department of Angiology and Vascular Surgery, Hospital do Divino Espírito Santo, Ponta Delgada, Azores, Portugal

**Keywords:** Acute care surgery, Giant cell arteritis, Temporal artery biopsy

## Abstract

Temporal artery biopsy (TAB) for suspected giant cell arteritis (GCA) represents an infrequent but important inpatient consultation for acute care surgery (ACS) services. Given the morbidity associated with delayed treatment, corticosteroids are often initiated before biopsy, raising questions about the diagnostic utility and impact of TAB in contemporary practice. We performed a review of all inpatient TABs completed by an ACS service at a tertiary center between March 2015 and January 2024 to characterize case volume, pathology results, and influence of biopsy findings on corticosteroid management.

Sixty-four patients underwent TAB, the majority of whom were elderly and female, with visual symptoms and headache being the most common presenting complaints. Nearly all patients were initiated on high-dose corticosteroids prior to biopsy, with a median of two days between ACS consultation and procedure. Pathologic findings confirmed GCA in 10% of cases, with additional biopsies demonstrating intimal fibroplasia or equivocal inflammatory changes. Despite widespread steroid initiation, biopsy results influenced discharge management, as patients with negative pathology were more likely to undergo corticosteroid tapering prior to or shortly after discharge. The median American College of Rheumatology 1990 classification score was three, with just over half of patients meeting criteria for GCA. No TABs were performed during 2020, but biopsy volume increased substantially in the post-COVID period.

These findings suggest that ACS services play an important role in the diagnostic evaluation of suspected GCA. Although corticosteroid therapy is frequently initiated prior to biopsy, TAB continues to yield clinically meaningful information that informs subsequent management decisions.

## Introduction

Temporal artery biopsy (TAB) to diagnose giant cell arteritis (GCA) is an occasional inpatient consultation for acute care surgery (ACS) services. GCA typically affects those of advanced age, and with a rapidly growing elderly population the demand for biopsies is likely to increase [1]. As the risks of missing a GCA diagnosis are significant, prompt diagnosis is critical to optimize patient outcomes. However, signs and symptoms such as headache, scalp tenderness, and jaw claudication; laboratory values such as erythrocyte sedimentation rate (ESR) and C-reactive protein (CRP); and even histological diagnosis with TAB have diagnostic sensitivity and specificity limitations [2].

Given the significant consequences of a missed diagnosis, treatment of GCA with high dose corticosteroids is started as early as possible to reduce risk of vision loss and to ameliorate symptoms. However, as most patients with suspected GCA have already started corticosteroid treatment before TAB has been undertaken or pathology results are available, TAB's main role may be in allowing patients to avoid prolonged courses of corticosteroids by ruling out, rather than confirming GCA diagnosis. Additionally, the time between corticosteroid initiation and TAB may influence arterial inflammation and pathology [3].

ACS services have been developed over the last two decades and cover a wide range of emergency and diagnostic surgical procedures. TAB is within this realm; however, there is long-standing debate among ACS surgeons regarding the procedure's utility in the diagnosis and management of suspected GCA despite specificity approaching 100% [4]. To date there has not been an examination of TABs completed by an ACS service. Our goal in this descriptive review is to assess TAB volume and recent trends on an ACS service, examine pathology results, and to determine how treatment plans were affected by these results.

## Methods

This is a retrospective chart review of all inpatient TAB completed by the ACS service at a major urban tertiary referral center from March 2015 through January 2024. IRB approval was obtained. Charts were reviewed manually by the research team(THF and AHS) from the time of their admission when the biopsy occurred through discharge. History and physical notes, consultant notes, operative reports, pathology reports and discharge summaries were reviewed for each patient. Patient demographics (sex; age at time of presentation) and symptoms common of GCA, including new visual symptoms (vision changes, blurriness, transient or permanent blindness), new headache or facial pain of any distribution, new jaw pain, and constitutional symptoms were recorded. The first-recorded erythrocyte sedimentation rate (Westergren method) (ESR) and C-reactive protein (CRP) laboratory values, as well as corticosteroid administration timing, were noted. Corticosteroid disposition on discharge was categorized as either: 1) stop corticosteroids on discharge; 2) begin outpatient taper of corticosteroids on discharge; 3) continue corticosteroids at current dose until outpatient rheumatology follow-up; or 4) no corticosteroids prescribed during or after hospitalization.

The 1990 American College of Rheumatology GCA Classification Criteria Score [5] was calculated for each patient; a score of three or more of the five classification criteria (age ≥ 50; new headache; temporal artery tenderness or decreased pulsation; ESR ≥50 mm/h; positive TAB pathology) diagnoses GCA with a sensitivity of 94% and specificity of 91%. Patient records did not reliably report temporal artery examination; thus, the reported score represents a conservative measure.

Finally, surgical pathology records were examined, noted for mentioning “intimal fibroplasia” or “acute vasculitis,” two common histological features associated with GCA [6], along with specimen length and date of report. Additional relevant commentary from the pathologist was also recorded. Data preparation and analysis were completed with Microsoft Excel and Stata 18.0 (StataCorp LLC, College Station, TX). This retrospective study was reviewed and approved by the Lifespan/Brown University Health Institutional Review Board with a waiver of informed consent.

## Results

64 patients (62.5% female, mean age 70.9) received TAB for suspected GCA (63 unilateral, 1 bilateral) (Table 1). The most common presenting symptoms were changes in vision (84.4%) and headache/facial pain (76.6%). 30% of patients had jaw claudication on presentation. Initial inflammatory markers varied widely (ESR median 64, IQR 46.5; CRP median 15, IQR 36).

**Table 1:** Temporal Artery Biopsy Patient and Case Characteristics.

	N (%)
All patients	64 (100)
Female	40 (62.5)
Steroid initiated prior to biopsy	62 (96.9)
	**Median (IQR)**
Age	71 (14)
Days from consult to surgery	2 (2)
Steroid days at biopsy	2 (2)
*Inflammatory markers on presentation*	
ESR	64 (46.5)
CRP	15 (36)
*Symptoms on presentation*	**N (%)**
Visual	54 (84.4)
Headache/facial pain	49 (76.6)
Jaw claudication	19 (29.7)
*Surgical pathology findings indicative of GCA*	
Intimal fibroplasia	13 (20.3)
Confirmed GCA	6 (9.7)
*American College of Rheumatology 1990 GCA Classification Score*	
Score Median (IQR)	3 (1)
Number of patients with score indicating GCA (score ≥ 3)	34 (53.1)

The median time from ACS consult to TAB was 2 days (IQR 2), and by the time of biopsy, 97% of patients were initiated on high-dose corticosteroids. Specimen pathology revealed 13 cases (20.3%) of intimal fibroplasia, 6 cases (10%) of confirmed GCA and 1 case of “other vasculitis,” along with 2 cases of equivocal/borderline GCA, 3 cases of “potentially healed arteritis,” and 2 cases of Mönckeberg's sclerosis. 43 patients (67.2%) were discharged on high-dose corticosteroids to be managed by outpatient rheumatology, while 18 (28.1%) were discharged with a taper plan. Of those discharged with a taper, 12 (66%) received biopsy results before discharge, all of which were negative for GCA. (Table 2) Of the 6 patients with positive pathology for GCA, 5 remained on corticosteroids till follow up and one patient was placed on a taper plan. This patient who was tapered was placed on steroids for presumed GCA three weeks before he was admitted for left eye and jaw pain. He then had a biopsy that admission that was positive but decision was made to taper and on chart review a month later he went on to have a recurrence and was readmitted and was treated with high dose corticosteroids and tocilizumab.

**Table 2:** Steroid decisions.

*Steroid plan on discharge*	
Discontinued	3 (4.7)
Continued until outpatient follow-up	43 (67.2)
Tapered (total, regardless of pathology)	18 (28.1)
Tapered with negative biopsy before hospital discharge	12 (66.6)
Tapered with negative biopsy after hospital discharge	6 (33.3)

The median 1990 American College of Rheumatology GCA Classification Criteria Score was 3 (IQR 1). 34 of the 64 patients in the study (53.1%) scored highly enough to be diagnosed with GCA per these 1990 guidelines.

**Fig. 1:**
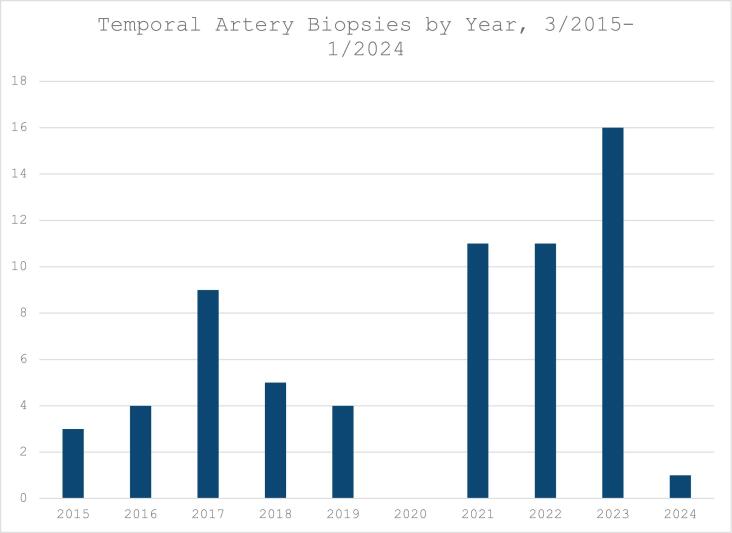
Temporal Artery Biopsies by Year, 2015–2024.

No TAB for suspected GCA were completed in 2020. The mean number of TAB increased in the post-COVID era to 13/year (excluding 2024), compared to 5/year pre-COVID (Fig. 1).

## Discussion

This study is the first to our knowledge to examine the experience of an ACS service in inpatient evaluation of GCA, a relatively uncommon consultation that our data suggests may be increasing in frequency. Despite the urgency to initiate treatment, the time between consultation and TAB calls into question the validity of undertaking the biopsy procedure. Our data show that nearly all patients are initiated on corticosteroid treatment by the time of TAB. The risk-benefit analysis of initial management of suspected GCA has long favored starting high-dose corticosteroids due to concern for development of irreversible vision loss and other adverse events. Given that our data show a median time from consultation to biopsy of 2 days, waiting to start corticosteroids for the biopsy is not reasonable. These data also affirm that even when corticosteroid therapy is initiated prior to biopsy, TAB still yields GCA-positive pathologic results in 10% of the studied patients, a proportion in line with recent work [7], [8], [9]. This further suggests the importance of ACS services in performing TAB for suspected GCA.

Recent guidelines from the European Society of Endocrinology suggest that a corticosteroid taper is only indicated after 3–4 weeks of corticosteroid therapy [10]. In this series, there exists possible opportunity for improvement in following up pathology and stopping corticosteroid treatment before discharge, as most patients had negative results by the time of hospital discharge. False negative results do occur though due to inadequate specimens and skip lesions, so routine rheumatology follow-up should be considered for all patients that underwent workup for GCA.

Other studies provide perspective to our findings. Ray-Chaudhuri performed a prospective comparative study of 11 patients that met ACR 1990 criteria for GCA [11]. The authors noted that there were no differences in rate of positive results whether the biopsies were done within 2 weeks or after 4 to 6 weeks of corticosteroid therapy. A cohort study at the Mayo Clinic of 535 patients found similar rates of positive specimens whether biopsy was done before or after corticosteroids were started, 31% versus 35%(*p* = 0.4) [12] Patients that received corticosteroids before biopsy had more clinical features of GCA but after logistic regression positivity rates on pathology were not related to timing of biopsy relative to corticosteroid commencement. More recently, Villeneuve et al. in a retrospective review of 127 patients found a 24% rate of positive specimens with jaw claudication to be highly associated with positivity [8]. Again, corticosteroid duration before biopsy was not associated with differences in positivity rates.

Studies reporting higher biopsy positivity rates, including those that prospectively applied ACR criteria, suggest opportunities to improve patient selection. Incorporating objective clinical criteria into ACS consultations may reduce unnecessary inpatient biopsies and improve diagnostic yield. These findings support a role for ACS surgeons not only in providing procedural access but also in contributing to diagnostic stewardship for patients with suspected giant cell arteritis. The ACS service maintained timely access to biopsy, with procedures performed a median of 2 days after consultation.

There are some important limitations of our study. This is a small study, 9 surgeons across 64 patients, where uniformity in approach is not guaranteed. Additionally, we were unable to determine from our dataset if certain surgeons were more selective in performing biopsies based on interpretation of clinical findings. This study would also benefit from ability to review outpatient follow up notes from primary care physicians and rheumatologists to understand decision making longer-term. Finally, the average number of TAB performed following the COVID-19 pandemic, compared to prior, suggests a pattern of increasing TAB prevalence that will likely continue with the rapidly growing advanced age population.

## Conclusions

ACS services are performing an increasing number of TAB for GCA. Despite diagnostic uncertainty of this condition before biopsy, this series suggests that corticosteroid administration prior to TAB does not significantly alter inflammation, and thus confound pathology results, within a median 2-day period between steroid initiation/surgical consultation and TAB. ACS services thus remain important in determining the diagnosis and subsequent management of suspected GCA**.**

## CRediT authorship contribution statement

**Tynan H. Friend:** Writing – original draft, Conceptualization. **Madeline Goosman:** Writing – review & editing, Data curation. **Abigail Hancock:** Data curation. **Adam Aluisio:** Writing – review & editing. **Nelson Oliveira:** Writing – review & editing, Validation. **Manuel Silva:** Writing – review & editing, Validation. **Emanuel. Dias:** Writing – review & editing, Validation, Supervision. **Daithi S. Heffernan:** Writing – review & editing, Validation, Supervision, Formal analysis. **Andrew H. Stephen:** Writing – review & editing, Data curation, Conceptualization.

## Ethics approval

This retrospective study was reviewed and approved by the Lifespan/Brown University Health Institutional Review Board, with a waiver of informed consent.

## Funding

This research did not receive any grant from funding agencies in the public, commercial, or not-for-profit sectors.

## Declaration of competing interest

No authors of this manuscript report any financial or personal conflicts of interest.
